# Tumor-Targeting Glycol Chitosan Nanoparticles for Image-Guided Surgery of Rabbit Orthotopic VX2 Lung Cancer

**DOI:** 10.3390/pharmaceutics12070621

**Published:** 2020-07-03

**Authors:** Kyeong Cheol On, Jiyun Rho, Hong Yeol Yoon, Hyeyoun Chang, Ji Young Yhee, Jun Sik Yoon, Seo Young Jeong, Hyun Koo Kim, Kwangmeyung Kim

**Affiliations:** 1 Department of Life and Nanopharmaceutical Sciences, Graduate School, Kyung Hee University, Seoul 02447, Korea; auncok@naver.com (K.C.O.); uuhu@kist.re.kr (J.S.Y.); syjeong@khu.ac.kr (S.Y.J.); 2 Center for Theragnosis, Biomedical Research Institute, Korea Institute of Science and Technology (KIST), Seoul 02792, Korea; seerou@kist.re.kr; 3 Department of Biomedical Sciences, College of Medicine, Korea University, Seoul 02841, Korea; jiyun.r1219@gmail.com; 4 Department of Thoracic and Cardiovascular Surgery, College of Medicine, Korea University Guro Hospital, Seoul 08308, Korea; 5 Department of Cancer Biology, Dana-Farber Cancer Institute, 450 Brookline Ave, Boston, MA 02215, USA; Hyeyoun_Chang@DFCI.HARVARD.EDU; 6 Pharosgen Co. Seoul 05505, Korea; yhee@pharosgen.co.kr; 7 KU-KIST Graduate School of Converging Science and Technology, Korea University, 145 Anam-ro, Seongbuk-gu, Seoul 02841, Korea

**Keywords:** glycol chitosan nanoparticle, image-guided surgery, tumor-targeted delivery

## Abstract

Theranostic nanoparticles can deliver therapeutic agents as well as diverse imaging agents to tumors. The enhanced permeation and retention (EPR) effect is regarded as a crucial mechanism for the tumor-targeted delivery of nanoparticles. Although a large number of studies of the EPR effect of theranostic nanoparticles have been performed, the effect of the change in the body size of the host on the EPR effect is not fully understood. In this regard, comparative research is needed on the behavior of nanoparticles in large animals for developing the nanoparticles to the clinical stage. In this study, we prepared fluorophore (indocyanine green (ICG) or cyanine 5.5 (Cy5.5))–conjugated glycol chitosan nanoparticles (CNPs) for comparing the tumor-targeting efficacy in VX2 tumor-bearing mouse and rabbit models. As expected, the CNPs formed nano-sized spherical nanoparticles and were stable for 8 days under aqueous conditions. The CNPs also exhibited dose-dependent cellular uptake into VX2 tumor cells without cytotoxicity. The half-life of the near-infrared fluorescence (NIRF) signals in the blood were 3.25 h and 4.73 h when the CNPs were injected into mice and rabbits, respectively. Importantly, the CNPs showed excellent tumor accumulation and prolonged biodistribution profiles in both the VX2 tumor-bearing mouse and rabbit models, wherein the tumor accumulation was maximized at 48 h and 72 h, respectively. Based on the excellent tumor accumulation of the CNPs, finally, the CNPs were used in the image-guided surgery of the rabbit orthotopic VX2 lung tumor model. The lung tumor tissue was successfully removed based on the NIRF signal from the CNPs in the tumor tissue. This study shows that CNPs can be potentially used for tumor theragnosis in small animals and large animals.

## 1. Introduction

Tumors have abnormal blood vessels that enable the extravasation and retention of nanoparticles, which can be utilized as a means of inducing the tumor accumulation of various nanomaterials after systemic administration [1]. This feature in tumors is well known as the enhanced permeation and retention (EPR) effect. The tumor microenvironment, exhibiting poor lymphatic drainage, high interstitial fluid pressure, and a dense extracellular matrix, plays a role in the EPR effect in the tumor tissue [2,3]. To date, a number of nanoparticles have been developed for delivering drugs and imaging agents to the tumor using the EPR effect, leading to significantly improved efficiency in both the therapy and diagnosis of the tumor [4]. The main advantages of nanoparticle-based drug delivery systems are (i) the prolonged circulation time of the drugs, (ii) improved accumulation in the tumor, (iii) enhanced cellular uptake, and (iv) reduced toxicity in healthy tissues. For these purposes, various nanoparticle strategies based on changes in structure, composition, or conformation have been adopted in nanoparticle-based drug delivery systems [5,6]. However, the EPR effect-based delivery of nanoparticles has been evaluated primarily using small animal models such as mice. The difference in the tumor to bodyweight ratio between mice and large animal tumor models may change the pharmacokinetics and delivery efficiency of nanoparticles [7,8]. Thus, the evaluation of the nanoparticle delivery effect using a large animal tumor model would provide a research strategy for the clinical translation of nanoparticles.

For tumor treatments, in vivo near-infrared fluorescence (NIRF) imaging techniques have contributed to better diagnosis and therapy over the past two decades. The NIRF is able to provide high-contrast images since its scattering and tissue auto-fluorescence is relatively low [9]. Therefore, NIRF has been utilized for different targets such as tumors [10,11,12], sentinel lymph nodes [13,14,15,16], cardiovascular diseases [17,18], and neurological diseases [19,20]. As small molecules, near-infrared (NIR) fluorescent molecules have been used as contrast agents in surgery since they can provide surgical guidance based on the EPR effect of tumor tissues [21]. In combination with the intraoperative imaging system, NIRF contrast agents have been utilized for open surgery [22], laparoscopy [23,24], and thoracoscopy [25] in clinical trials [26]. In particular, image-guided surgery has shown great potential in enhancing the successful surgical removal of tumors by enabling the visualization of the margins between tumors and normal tissues in real time. For image-guided surgery, NIR fluorescent molecules such as indocyanine green (ICG), with emission and excitation wavelengths ranging from 700–900 nm, have been utilized to guide surgeons, thereby allowing the precise removal of tumors [9,10]. However, the biodistribution and clearance rates of NIR fluorescent molecules vary drastically depending on the type of the fluorescent molecules [27], and NIR fluorescent molecules generally circulate in the bloodstream for only a short period of time [28]. To address these limitations, various designs of NIR fluorescent molecules using peptides, proteins, and antibodies and nanoparticles have been developed for the delivery of NIR fluorescent molecules [29].

Glycol chitosan (GC) is a derivative of chitin in which is incorporated a hydrophilic glycol group and that is partially deacetylated in the backbone. The abundant functional groups of GC allow easy fabrication via chemical modification with anticancer agents and hydrophobic molecules [30]. In particular, hydrophobically modified GC can form self-assembled nanoparticles (CNPs), and the hydrophobic inner site can be used for the encapsulation of various theranostic agents. These CNPs showed prolonged blood circulation and high tumor accumulation via the EPR effect in tumor tissue, resulting in the facilitation of the efficient delivery of anticancer agents as well as imaging agents in preclinical mouse tumor models [31].

Herein, we prepared fluorophore (indocyanine green (ICG) or cyanine 5.5 (Cy5.5))-conjugated CNPs to compare tumor accumulation in VX2 tumor-bearing mice and rabbit models. The CNPs were successfully formed nanoparticles that could effectively accumulate in the tumor tissue by the EPR effect. Despite the differences in the pharmacokinetic properties of the CNPs, the NIRF signals from the CNPs could be used to effectively visualize tumor sites in both mouse and rabbit VX2 tumor models. Interestingly, we found that the VX2 tumors of the mouse and rabbit models showed strong similarities in the tumor accumulation of the CNPs as well as similar distributions in the organs after intravenous injection. Finally, we applied CNPs as an imaging contrast agent in the image-guided surgery of an orthotopic rabbit VX2 lung tumor model. CNPs could provide more stable and enhanced tumor-specific NIRF signals than free ICG during the image-guided surgical removal of tumor tissues in the rabbit orthotopic VX2 lung tumor model. CNPs can provide advantages for tumor-targeted imaging as well as the image-guided surgery of tumors from the mouse tumor model and rabbit tumor model.

## 2. Materials and Methods

### 2.1. Materials

Glycol chitosan (GC; molecular weight, 2.5 × 10^5^ Da; the degree of deacetylation, 82.7%), 5β-cholanic acid, N-hydroxysuccinimide (NHS; purity, >99%), 1-ethyl-3-(3-dimethylaminopropyl)-carbodiimide hydrochloride (EDC; purity, >99%), methanol (purity, >99%), and anhydrous dimethyl sulfoxide (DMSO; purity, >99%) were purchased from Sigma Aldrich (St. Louis, MO, USA). The mono-functional hydroxysuccinimide ester of cyanine 5.5 (Cy5.5-NHS) and indocyanine green (ICG-NHS) were purchased from GE Healthcare Life Sciences (Chicago, IL, USA) and Bioacts (Incheon, Korea), respectively. For the cell experiments, the Roswell Park Memorial Institute 1640 (RPMI-1640) medium, Dulbecco’s phosphate buffered saline (DPBS), fetal bovine serum (FBS), antibiotics (penicillin with streptomycin), and trypsin–EDTA were purchased from Welgene. Inc. (Daegu, Korea). All the other chemicals were purchased at reagent grade and used without further purification.

### 2.2. Preparation and Characterization of Glycol Chitosan Nanoparticles (CNPs)

Glycol chitosan nanoparticles (CNPs) were prepared as described in a previous report [32]. Briefly, GC (500 mg) was dissolved in a methanol/distilled water cosolvent (120 mL, 50:50 *v/v*) and mixed with N-hydroxysuccinimide (NHS, 72 mg) and 1-ethyl-3-(3-dimethyl laminopropyl)-carbodiimide hydrochloride (EDC, 120 mg). 5β-cholanic acid (150 mg/120 mL methanol) was added dropwise to GC solution for forming amide bonds between the GC and 5β-cholanic acid. Then, the solution was vigorously stirred for 24 h at room temperature. Unreacted 5β-cholanic acid, EDC, and NHS were removed by dialysis against distilled water/methanol (1:3, 1:1, and 1:0 *v/v*; molecular weight cut-off = 12–14 kDa) and the remaining contents lyophilized to obtain white powder, the CNPs. The chemical structure of the CNPs was confirmed using 600 MHz ^1^H-NMR (DMSO-d_6_, DD2 600 MHz FT NMR, Agilent Technologies, Santa Clara, CA, USA). For monitoring the distribution of CNPs in vitro and in vivo, 20 mg of CNPs were dissolved in 10 mL of DMSO, and 1 *wt*% of Cy5.5-NHS or ICG-NHS was mixed with the CNP solution to label the CNPs, respectively. The unreacted Cy5.5-NHS or IGC-NHS molecules were removed by dialysis against distilled water for 2 days (molecular weight cut-off = 12–14 kDa). The final Cy5.5-CNP or ICG-CNP solutions were lyophilized for 2 days. The lyophilized material was dispersed in sterile phosphate-buffered saline (PBS, pH 7.4) to build up self-assembled GC nanoparticles (CNPs) using a probe-type sonicator. The size distribution and stability (at 37 °C for 8 days) of the Cy5.5-CNPs and ICG-CNPs were measured using a dynamic light scattering system (DLS, Malvern Instruments, Sutton Coldfield, UK). The morphologies of the Cy5.5-CNPs and ICG-CNPs were measured using a transmission electron microscope (TEM; Tecnai F20, FEI, Netherlands) after negative staining using 2% uranyl acetate. The NIRF signals from the Cy5.5-CNPs and ICG-CNPs were monitored at various concentrations (0.6, 1.25, and 2.8 mg/mL in the distilled water) using the IVIS Spectrum imaging system (PerkinElmer, Waltham, MA, USA).

### 2.3. Cellular Uptake and Cytotoxicity of CNPs

For the cellular experiments, VX2 tumor cells were isolated from rabbit VX2 tumor tissues using a Cancer Cell Isolation Kit (Panomics Inc.,Fremont, CA, USA) [33]. In brief, the VX2 tumor tissue was washed using 10 mL of RPMI-1640 medium (with 1% penicillin/streptomycin), and non-tumor tissues and necrotic tumor tissues were eliminated from the tumor tissue. Then, small pieces of tumor tissue were suspended in 20 mL of RPMI-1640 medium and centrifuged at 1200 rpm for 6 min at room temperature. The pellet was dispersed and incubated with 10 mL of tumor cell digestion solution for 3 h at 37 °C. After digestion, 10 mL of cell suspension solution was added into the digestion solution, followed by the elimination of tissue residue using a 100 mm cell strainer. Then, the cells were washed twice with 20 mL of suspension solution by centrifugation at 1200 rpm for 8 min. For the purification of the tumor cells, cell suspension solution was slowly transferred to the tumor-cell purification solution (20 mL) and separated for 6 min at room temperature. Finally, 5 mL of the purification solution from the bottom, which contained tumor cells, was centrifuged at 1200 rpm for 8 min to collect the VX2 tumor cells. The isolated tumor cells were incubated with RPMI-1640 (with 10% FBS, 1% penicillin/streptomycin) for 5 days, and the morphology of isolated cells was monitored. For the observation of the cellular uptake of the CNPs, 1 × 10^5^ VX2 tumor cells were seeded into a 35 mm cover glass-bottom dish, and the cells were stabilized for 24 h at 37 °C in a CO_2_ incubator. Then, the tumor cells were incubated with 0, 50, 100, and 200 μg/mL solutions of Cy5.5-CNP-containing RPMI1640 medium for 24 h at 37 °C. The VX2 tumor cells were washed twice with cold DPBS and fixed using a fixation solution (2% formaldehyde–glutaraldehyde solution) for 10 min. Then, the VX2 tumor cells were washed twice with DPBS and mounted with a DAPI-contained mounting solution. Finally, the fluorescence signals from the VX2 tumor cells were observed using a confocal laser microscope (Leica TCS SP8, Leica Microsystems GmbH, Wetzlar, Germany). To measure the cytotoxicity of the CNPs to the VX2 tumor cells, the VX2 tumor cells were seeded onto 96-well plate (5 × 10^3^ cells/well) and stabilized for 24 h at 37 °C. Then, the VX2 tumor cells were incubated with various concentrations (12.5 to 100 μg /mL) of CNPs, Cy5.5-CNPs, and ICG-CNPs for 24 h at 37 °C After incubation, the cells were washed twice using DPBS, and then, the Cell Counting Kit-8 solution (10 μL, Dojindo Molecular Technologies Inc., Tabaru, Japan) was added into each well. Finally, the VX2 tumor cells were further incubated for 2 h at 37 °C, and the absorbance at 450 nm was recorded using a microplate reader (VERSAmaxTM, Molecular Devices Corp., Sunnyvale, CA, USA).

### 2.4. Pharmacokinetic Analysis of CNPs Using Mouse and Rabbit Blood

All the experiments with live animals were performed in compliance with the relevant laws and institutional guidelines of the Institutional Animal Care and Use Committee (IACUC) in the Korea Institute of Science and Technology (KIST), and IACUC approved the experiment (approval number of 2017-005 (KIST), 21 Dec 2017, KOREA-2016-0044, (Korea University), 24 May 2017). For the pharmacokinetic analysis of CNPs after administration to mouse and rabbits, 22.5 mg/kg of ICG-CNPs were injected through the tail veins of Balb/c mice (*n* = 5, 5-week old males) and ear vessels of New Zealand white rabbits (*n* = 2, 5-week old males). Then, 1 mL blood samples were collected at pre-determined time points. The blood samples were centrifuged at 12,000 rpm for 20 min at 4 °C. The NIRF intensity of the supernatants was measured using the IVIS Spectrum imaging system (PerkinElmer, Waltham, MA, USA) and quantified using the Living Image^®^ software 4.0 (Perkin Elmer, Waltham, MA, USA).

### 2.5. In Vivo Tumor Accumulation of CNPs in the Mouse and Rabbit VX2 Tumor Models

To establish mouse subcutaneous flank tumor models, five-week-old female athymic nude mice (*n* = 3; Orient Bio, Seoul, Korea) were anesthetized and injected with VX2 rabbit squamous cell carcinoma cells (1 × 10^6^ cells/80 µL PBS) subcutaneously over the left flank. The mice were observed for approximately five weeks until the tumors grew to a size of 400–500 mm^2^. To establish rabbit tumor models, rabbits were anesthetized with a cocktail of Tiletamine-Zolazepam 50 (Zoletil; 10 mg/kg; Virbac Korea Inc., Seoul, Korea) and xylazine (Rompun; 5 mg/kg; Bayer Korea Inc., Seoul, Korea) via intramuscular injection. The VX2 tumor tissues from the rabbit donors were finely chopped and filtered through a 100 µm cell strainer to isolate the tumor cells. Then, VX2 tumor cell suspension (300 µL, 1 × 10^7^ cells/mL DPBS) was injected subcutaneously underneath three of the nipples (*n* = 3) to establish a subcutaneous xenograft model for in vivo imaging. When the tumors of the mouse model grew up to 300–400 mm^3^, 22.5 mg/kg of Cy5.5-CNPs or ICG-CNPs were intravenously injected into the VX2 tumor-bearing mice (*n* = 3) via the tail veins. The real-time in vivo distributions of Cy5.5-CNPs and ICG-CNPs were observed using the IVIS Spectrum imaging system (PerkinElmer, Waltham, MA, USA) and a custom-made intraoperative color and fluorescence imaging system (ICFIS) [34], respectively. The whole-body NIRF images were acquired up to 96 h post injection. At 96 h post injection, tumors and major organs (livers, spleens, lungs, kidneys, and hearts) were excised. The NIRF intensity of the tumors and major organs was measured using the IVIS Spectrum imaging system (PerkinElmer, Waltham, MA, USA) and quantified using the Living Image^®^ software (Perkin Elmer, MA, USA). For the observed whole-body NIRF images for the VX2 rabbit model, 22.5 mg/kg of ICG-CNP and free ICG (2 mg/kg, equivalent amount of ICG) were administered intravenously into the marginal ear veins of the subcutaneous VX2 tumor (approximately 500–600 mm^3^)-bearing rabbits under anesthesia. Real-time fluorescence images overlaid with white light were obtained using an ICFIS for up to 96 h post injection. After the completion of the in vivo imaging, the rabbits were euthanized via the injection of 10 mL of air into an ear vein, and the tumors and major organs were removed for additional ex vivo NIRF imaging with the IVIS Spectrum imaging system.

### 2.6. Image-Guided Surgery of Rabbit Orthotopic VX2 Lung Tumors after CNP Injection

For the establishment of the rabbit orthotopic VX2 lung tumor model, the VX2 tumor cell/matrigel/lipiodol mixture was directly injected to the lateral chest region using computed tomography (CT, Brillance 64; Philips, Amsterdam, The Netherlands)-guided interventional techniques [35]. In brief, a 26-gauge needle was inserted into the peripheral lung region under the guidance of real-time CT fluoroscopy images after the administration of local anesthesia. Then, the VX2 tumor cell/matrigel/lipiodol mixture (1:1:1; <450 µL total volume) was injected into the lung parenchyma without pneumothorax or hemothorax. At 12 h post inoculation, the rabbits received the antibiotic enrofloxacin (10 mg/kg; Daewoong Pharmaceutical Co., Seoul, Korea) via intramuscular injection. Four weeks after tumor inoculation, 22.5 mg/kg of ICG-CNPs and ICG alone (2 mg/kg, equivalent amount of ICG) were administered intravenously under anesthesia. A mechanical ventilator connected to a tracheostomy ensured a continuous supply of 2% isoflurane. Vital signs were monitored throughout the surgery by a clinical anesthetist. The image-guided surgical removal of the primary tumor was performed at 72 h post ICG-CNP administration. Real-time white light-overlaid fluorescence imaging of the surgical site was performed using a custom-made ICFIS. After the surgery, the rabbits were sacrificed by injecting air into an ear vein. Subsequently, the surgically removed VX2 tumor tissues were fluorescently imaged using the IVIS Spectrum imaging system.

### 2.7. Tissue Fluorescence Analysis

To observe tissue fluorescence, excised tissues were fixed with OCT compound and frozen for 24 h at −20 °C. Then, the tissue block was sectioned at 10 µm and stained with DAPI. The fluorescence of the mounted tissue slides was monitored using a confocal laser microscope (Leica TCS SP8, Leica Microsystems GmbH, Germany) with 405 diode (405 nm), Ar (488, 514 nm), and He-Ne (633 nm) lasers.

### 2.8. Statistical Analysis

In this study, all the data are presented as the mean ± standard deviation. The statistical differences between the experimental and control groups were analyzed using one-way ANOVA in the Origin 2020 software (OriginLab Corporation, Northampton, MA, USA) and considered statistically significant if marked with an asterisk (*) in the figures.

## 3. Results and Discussion

### 3.1. Preparation and Characterization of Glycol Chitosan Nanoparticles (CNPs)

**Figure 1:**
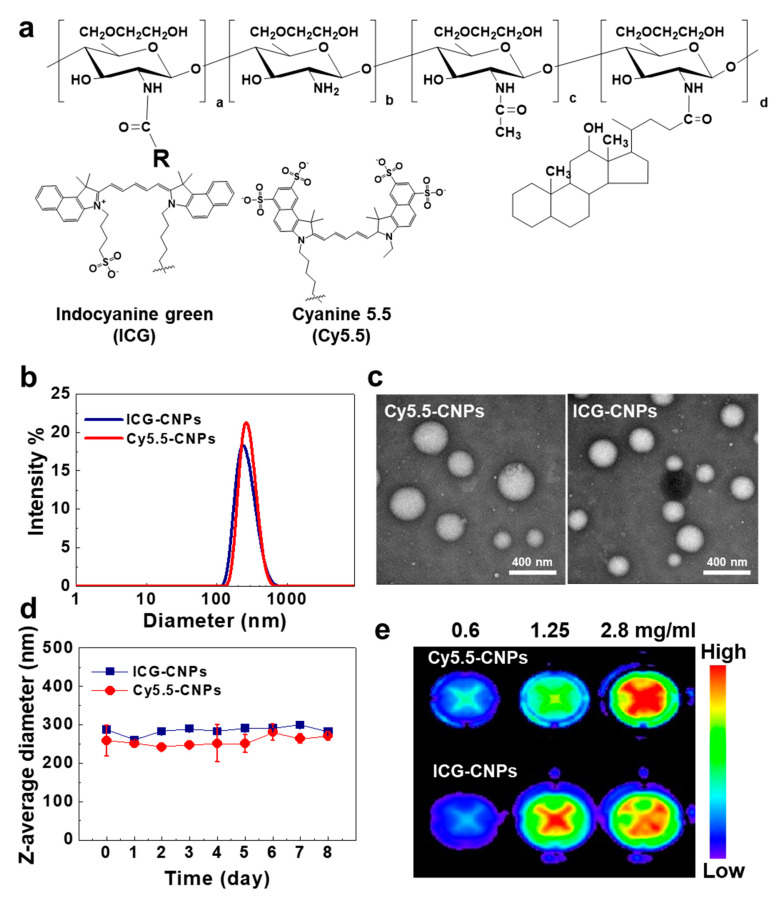
Preparation and in vitro characterization of cyanine 5.5 (Cy5.5) or indocyanine green (ICG)-conjugated glycol chitosan nanoparticles (CNPs). (**a**) Chemical structures of Cy5.5-CNPs and ICG-CNPs. (**b**) The size distributions of Cy5.5-CNPs and ICG-CNPs. (**c**) The TEM images of Cy5.5-CNPs and ICG-CNPs. The scale bar indicates 400 nm. (**d**) The size stability of Cy5.5-CNPs and ICG-CNPs. (**e**) The near-infrared fluorescence (NIRF) signals at various concentrations (0.6 to 2.8 mg/mL) of Cy5.5-CNPs and ICG-CNPs.

CNPs were prepared by directly conjugating 5β-cholanic acid to the amine group of GC in the presence of EDC and NHS. The degree of substitution of 5β-cholanic acid in the CNPs was calculated at 11% by characteristic peaks at 0.69, 0.93, 0.95, and 1–2.5 ppm of the ^1^H-NMR spectrum (Supporting Figure S1) [36]. For monitoring the in vitro and in vivo properties of the CNPs, Cy5.5 and ICG were covalently attached to the CNPs, respectively, to label them (Figure 1a). The amounts of Cy5.5 and ICG in the CNPs were calculated at 5.8 ± 0.2 *wt*% and 7.8 ± 0.4 *wt*% by using the absorbance peaks at 675 nm and 780 nm in the UV-Vis spectrum, respectively. Cy5.5-CNPs and ICG-CNPs showed similar hydrodynamic diameters that were 264 ± 3 nm and 261.8 ± 2.69 nm on average, respectively, with a unimodal size distribution (Figure 1b). The TEM images of the Cy5.5-CNPs and ICG-CNPs showed nano-sized spherical structures (Figure 1c). This is because CNPs can form self-assembled nanoparticles under physiological conditions due to their amphiphilic structure. The sizes of the Cy5.5-CNPs and ICG-CNPs were stably maintained for 8 days under physiological conditions (Figure 1d). Furthermore, the NIRF signals from the Cy5.5-CNPs and ICG-CNPs were gradually increased by increasing the concentration, indicating that fluorescent dyes were stably conjugated to the CNPs (Figure 1e). Based on these results, we expect that both Cy5.5-CNPs and ICG-CNPs have similar physicochemical properties and can be used for further in vitro and in vivo studies.

### 3.2. Cellular Uptake and Cytotoxicity of CNPs

**Figure 2:**
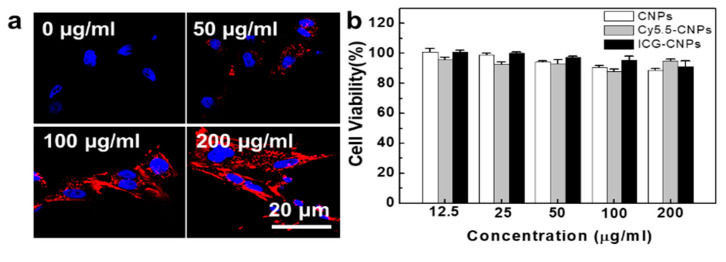
In vitro cellular uptake and cytotoxicity of CNPs in VX2 tumor cells. (**a**) Confocal microscope images of VX2 tumor cells after treatment with Cy5.5-CNPs (0 to 200 μg/mL). Blue channel = DAPI, red channel = Cy5.5-CNPs. (**b**) Cell viability of VX2 tumor cells treated with CNPs, Cy5.5-CNPs, and ICG-CNPs for 24 h. (*n* = 5).

To observe the cellular uptake and cytotoxicity of the CNPs in the cell culture system, the VX2 tumor cells were freshly isolated from the VX2 tumor tissue of the rabbit donor [33]. The isolated VX2 tumor cells were further cultured for 5 days, and the morphology of cells was recorded to compare it with that in the literature. The VX2 tumor cells were spindle-to-stellate-shaped cells that could be mono- or multi-nucleated, showing shapes similar to those in the literature (Supporting Figure S2) [37]. The confocal microscope images of the VX2 tumor cells showed that the NIRF signal of the Cy5.5-CNPs in the cytoplasm was gradually increased by the treatment concentration of Cy5.5-CNPs at 24 h (Figure 2a). This is because the Cy5.5-CNPs could internalize into the cells via a nanoparticle-derived cellular uptake mechanism [38,39]. To evaluate the cytotoxicity of Cy5.5-CNPs and ICG-CNPs in the VX2 tumor cells, the cell viability of the VX2 tumor cells that were treated with CNPs, Cy5.5-CNPs, and ICG-CNPs for 24 h was measured using Cell Counting Kit-8 (CCK-8). At the high concentration of 200 μg/mL, Cy5.5-CNP- and ICG-CNP-treated VX2 tumor cells did not show significant changes in cell viability compared to the CNP-treated VX2 tumor cells at 24 h (Figure 2b). These results showed that CNPs could be taken up by the VX2 tumor cells without cytotoxicity in the cell culture system.

### 3.3. Pharmacokinetic Analysis of CNPs Using the Mouse and Rabbit Blood

**Figure 3:**
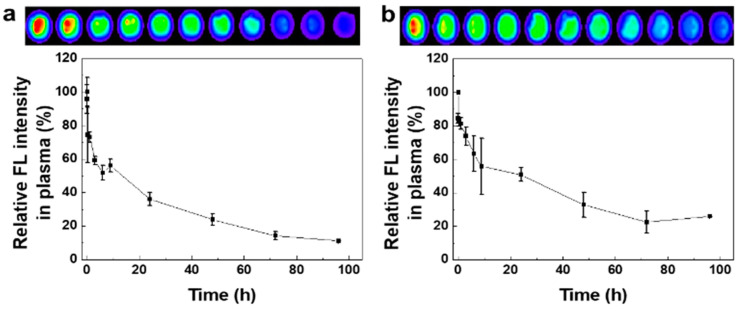
The pharmacokinetic analysis of ICG-CNPs using rabbit serum (*n* = 3) and mouse serum (*n* = 3). The NIRF signals from (**a**) mouse and (**b**) rabbit blood. The half-life of NIRF signals was calculated at 3.25 ± 0.36 h and 4.73 ± 0.2 h in mouse blood and rabbit blood, respectively. (*n* = 3, mean ± SD).

Prior to evaluating the in vivo tumor accumulation of CNPs, we compared the pharmacokinetic (PK) properties of CNPs in both normal mice and rabbits. The PK analysis of nanoparticles in the blood can help to predict the stability and circulation time of nanoparticles in the body, from which can be inferred the tumor accumulation [40,41]. For the PK analysis, 22.5 mg/kg of ICG-CNPs was injected into the mice and rabbits through the tail veins and ear veins, respectively. At 1 min, 15 min, 30 min, 1 h, 3 h, 6 h, 9 h, 24 h, 48 h, 72 h, and 96 h post injection, the blood was collected and centrifuged to separate the serum and platelets. The NIRF signals in the serum from both the mice and rabbits showed gradual decreases over time. The half-lives (T_1/2_s) of the NIRF signals were 3.25 h and 4.73 h when the ICG-CNPs were injected into the mice and rabbits, respectively (Figure 3a,b). This is because smaller animals have faster clearance per body weight [42]. Compared to the NIRF signals at 1 min, further 11.2% and 25.8% increases in the NIRF signals were observed in the mouse and rabbit sera at 96 h post injection, respectively. These results imply that ICG-CNPs could have a prolonged circulation time in the blood of the mouse as well as in that of the rabbit, which might be related to the increased tumor accumulation in both the mice and rabbits. Therefore, we expect that the CNP-based imaging probe could change the limited PKs of free ICG, which has a relatively short half-life of 2 to 4 min in the body, resulting in an improvement in tumor accumulation and the provision of high imaging sensitivity at the tumor [43].

### 3.4. In Vivo Tumor Accumulation of CNPs in the Mouse VX2 Tumor Model

**Figure 4:**
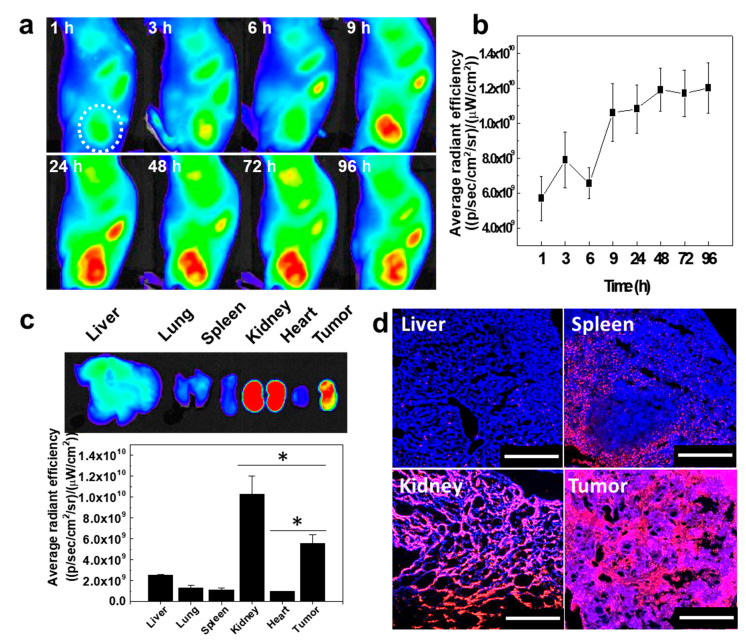
In vivo biodistribution of Cy5.5-CNPs in the VX2 tumor-bearing mouse (*n* = 3). (**a**) Whole-body NIRF images of Cy5.5-CNPs. White dotted circle indicates tumor tissue. (**b**) Quantified NIRF signals of Cy5.5-CNPs over time. (**c**) Ex vivo NIRF images and quantified NIRF signals from tumor tissue and organs. (*) indicates difference at the *p* < 0.01 significance level. (**d**) Tissue fluorescence images of liver, spleen, kidney, and tumor. Blue channel = DAPI, red channel = Cy5.5-CNPs. The scale bar indicates 250 µm.

Prior to the observation of the tumor accumulation of CNPs in the rabbit, the biodistribution and tumor accumulation of CNPs were monitored using the mouse VX2 tumor model. Cy5.5-CNPs (22.5 mg/kg) were injected via the tail vein of the VX2 tumor-bearing mouse. The accumulation of Cy5.5-CNPs in the tumor tissue was monitored by using the NIRF imaging system for 96 h. The NIRF signals in the tumor tissue were gradually increased for 24 h and saturated at 48 h (Figure 4a). Between 48 h and 96 h, the maximum signal intensities were measured in the tumor, indicating that the Cy5.5-CNPs could circulate in the body for a long time and effectively accumulate in the tumor (Figure 4b). The NIRF signals of the Cy5.5-CNPs in the major organs and tumor were observed at 96 h post injection (Figure 4c). Ex vivo NIRF imaging showed strong NIRF signals in both the tumor and kidneys. This is because GC derived from chitosan can bind to the megalin receptor, which is expressed on renal tubular cells, leading to the uptake and retention of CNPs in the kidney [44,45]. Furthermore, the strong NIRF signal of Cy5.5-CNPs in the kidneys implied that Cy5.5-CNPs could be excreted via the kidneys, which are the major organs that clear nanoparticles from the body [46]. Therefore, we expect that Cy5.5-CNPs have safe a PK profile. Notably, the NIRF signal of the tumor tissue was 2.2-, 4.22-, 4.9-, and 5.6-fold higher than the signals of the liver, lung, spleen, and heart, respectively. Furthermore, small amounts of nanoparticles were localized in liver, due to the minimal non-specific accumulation of Cy5.5-CNPs in the liver tissue. The biodistribution of ICG-CNPs in the mouse tumor models was also evaluated using VX2 tumor-bearing mice. The VX2 tumors on the left flank of the mice injected with the ICG-CNPs emitted signals under a fluorescence imaging system (ICFIS) for up to 96 h post injection (Supporting Figure S3a). The ex vivo NIRF analysis showed that the ICG-CNPs were distributed in the tumor, kidneys, and liver (Supporting Figure S3b). In particular, the most intense NIRF signals were mainly observed in the tumors, implying that ICG-CNPs specifially accumulated in the tumor tissue. Finally, tissue fluorescence images of the liver, spleen, kidneys, and tumor showed that strong NIRF signals could be observed in both the tumor tissue and kidney tissue (Figure 4d). Based on these results, we expected that CNPs have the potential to be used in fluorescence image-guided tumor removal surgery.

### 3.5. Image-Guided Surgery of Rabbit Orthotopic VX2 Lung Tumor after ICG-CNP Injection

**Figure 5:**
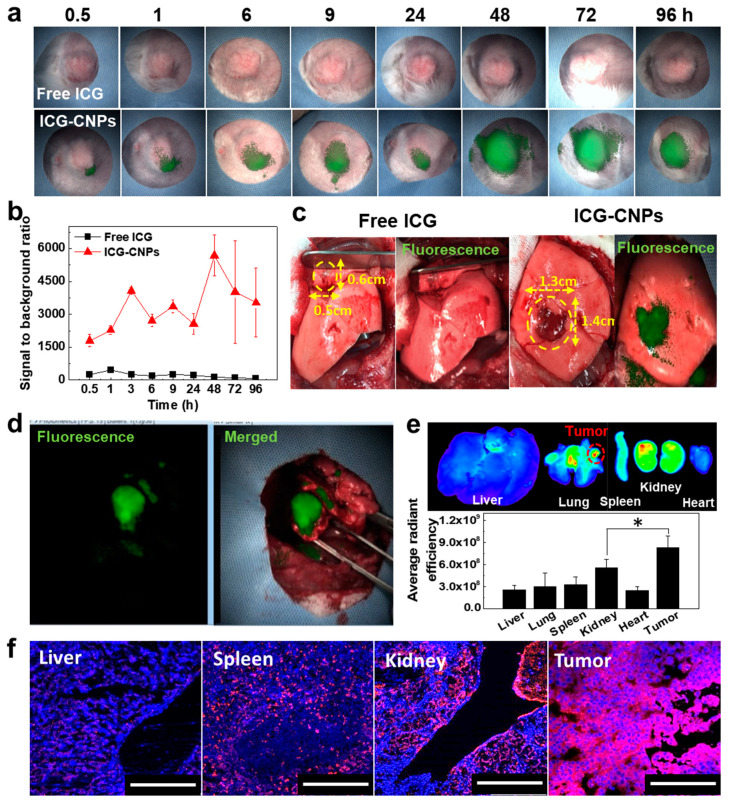
Tumor accumulation of free ICG and ICG-CNPs in rabbit VX2 tumor models (*n* = 3) and image-guided surgical removal of orthotopic VX2 lung tumors in rabbit models (*n* = 3). (**a**) Non-invasive NIRF imaging of VX2 tumors using ICFIS. (**b**) Signal to background ratios from (**a**). (**c**) White light-overlaid NIRF images of the surgical region at 96 h after the injections of free ICG and ICG-CNPs. All the data show differences at the *p* < 0.01 significance level. (**d**) The monitoring of the ICFIS instrument during the surgery. (**e**) Ex vivo NIRF images and signals in major organs and tumor tissue. Red dotted circle indicates tumor site. (*) indicates difference at the *p* < 0.05 significance level. (**f**) Tissue fluorescence images of liver, spleen, kidney, and tumor from orthotopic VX2 lung tumor model at 96 h post injection of ICG-CNPs.

Firstly, 22.5 mg/kg of ICG-CNPs and 2 mg/kg of free ICG were intravenously injected into subcutaneous VX2 tumor-bearing rabbits to evaluate the possibility of using the real-time NIRF imaging of ICG-CNPs as a tumor removal surgery guide. The VX2 tumors were monitored under an ICFIS for 96 h after ICG-CNP or free ICG injection (Figure 5a). The NIRF signals were observed at the tumor sites of the ICG-CNP-injected rabbits, which were gradually increased up to 48 h then saturated at 96 h. The tumors were more clearly differentiated with higher contrast to the background. Stable and intense NIRF signals suitable for image-guided surgery were observed until 96 h post injection. However, the tumors of the rabbits injected with 2 mg/kg of ICG alone showed only insignificant NIRF signals. The intensity of the ICG signal was slightly increased during the first 30 min after the injection. However, the signal intensity conspicuously decreased for the next 30 min, due to the rapid clearance profile of the free ICG (Figure 5b). Next, we performed real-time NIRF imaging and resection by thoracotomy after the injection of ICG-CNPs into the rabbit orthotopic VX2 lung cancer models, wherein the VX2 tumor cell mixture was directly injected to the lung parenchyma. At 96 h after the injection of the ICG-CNPs into the rabbit orthotopic VX2 lung tumor models, the real-time white light-overlaid fluorescence images showed clear margins of the tumors, distinguishing them from the non-cancerous lung tissues. In addition, high-contrast tumor imaging for the surgery was available for the tumors sized 1.3 × 1.4 × 1.0 cm^3^. On the contrary, no NIRF signal in the tumor tissue could be observed when the free ICG was injected into the rabbit orthotopic VX2 lung tumor models (Figure 5c).

Finally, the image-guided surgical removal of orthotopic VX2 lung tumors in rabbit models was performed based on the real-time NIRF signal from ICG-CNPs in the tumor tissue (Supporting Figure S4a). The real-time NIRF signal could easily distinguish the tumor’s margins with normal lung tissue, resulting in the facilitation of the removal of the tumor tissue from the lung (Figure 5d). The excised tumor tissue also showed tumor margins, indicating that the tumor tissue was successfully removed from the lung (Supporting Figure S4b and Supporting Movie). The ex vivo biodistribution of the ICG-CNPs between the major organs and tumor was observed at 96 h post injection (Figure 5e). An ex vivo NIRF image of the ICG-CNP-injected rabbit exhibited a strong NIRF signal in both the tumor and kidneys, which was similar in the ex vivo NIRF image of the VX2 mouse model. In particular, the strongest NIRF signal was observed in the tumor tissue that was located in the lung. Furthermore, the tissue fluorescence images of the liver, spleen, kidneys, and tumor showed NIRF signals, and the strongest NIRF signal was observed in tumor tissue (Figure 5f). Based on these results, the ICG-CNPs exhibited high tumor accumulation in the rabbit as well as in the mouse model, facilitating long-lasting high-contrast imaging. This is because the tumor accumulation and retention of ICG-CNPs, which have a size range of 250–270 nm, can be enhanced compared to that of the NIR fluorescent dye ICG due to the inherent property of CNPs. This property of ICG-CNPs would be especially useful in longer surgeries for the removal of multiple or complex tumors.

## 4. Conclusions

In this study, we prepared Cy5.5-CNPs and ICG-CNPs, excellent tumor-targeting nanoparticles, for image-guided surgery. Both the Cy5.5-CNPs and ICG-CNPs successfully accumulated in the tumor tissue, resulting in the visualization of the separation between tumors and normal tissue in both mouse and rabbit VX2 tumor models. During the image-guided surgical removal of an orthotopic VX2 lung tumor, the ICG-CNPs could provide more stable and long-lasting tumor-specific signals for NIRF imaging than free ICG, owing to the intrinsic properties of CNPs. Furthermore, it was found that the VX2 tumors of the mouse and rabbit models showed strong similarities in terms of the tumor accumulation of CNPs as well as showing similar ex vivo biodistributions after the injection of CNPs. Finally, CNPs can be potentially used as a theranostic platform for tumor treatment in a mouse tumor model and rabbit tumor model, as they demonstrated comparable efficiency in tumor-targeted delivery and imaging efficacy that effectively supported image-guided surgery.

## Supplementary Materials

The following are available online at https://www.mdpi.com/1999-4923/12/7/621/s1. Figure S1: 600 MHz ^1^H-NMR spectra; Figure S2: Morphologies of isolated VX2 tumor cells; Figure S3: Biodistribution of ICG-CNPs in VX2 tumor-bearing mouse; Figure S4: Representative images of image-guided surgery of VX2 tumor; Video S1: Image-guided surgery of VX2 tumor.
