# Supplementary material for: Tumor-Targeting Glycol Chitosan Nanoparticles for Image-Guided Surgery of Rabbit Orthotopic VX2 Lung Cancer
Source: Pharmaceutics. 2020 Jul 3;12(7):621. doi: 10.3390/pharmaceutics12070621 (PMC7407595; doi:10.3390/pharmaceutics12070621)
Supplement: Supplementary file 1 [file pharmaceutics-12-00621-s001.zip › pharmaceutics-846159 SP/pharmaceutics-846159-sp formatted.docx]

Supplementary Materials: Tumor-Targeted Glycol Chitosan Nanoparticles for Image-guided Surgery of Rabbit Orthotopic VX2 Lung Cancer

Kyeong Cheol On, Jiyun Rho, Hong Yeol Yoon, Hyeyoun Chang, Jun Sik Yoon, Seo Young Jeong, Hyun Koo Kim and Kwangmeyung Kim


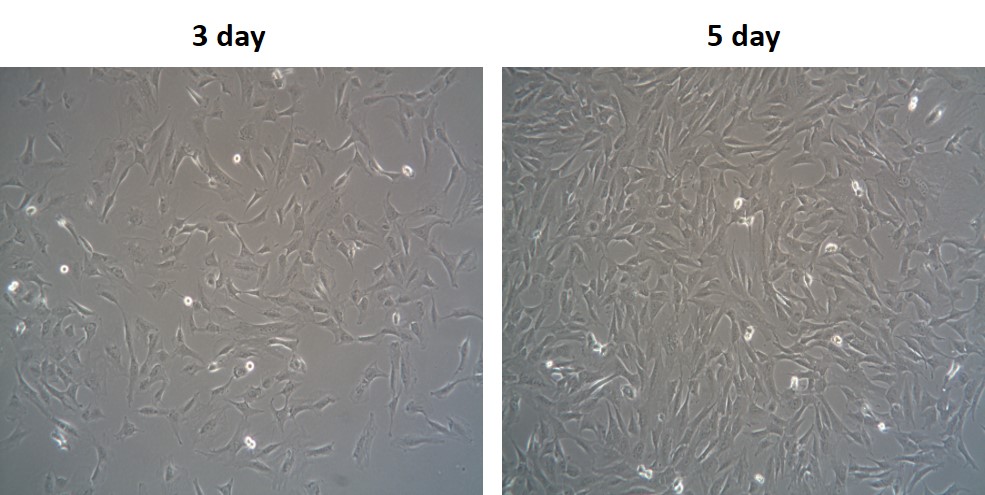


**Figure S1.** Morphologies of isolated VX2 tumor cells using cancer cell isolation kit.


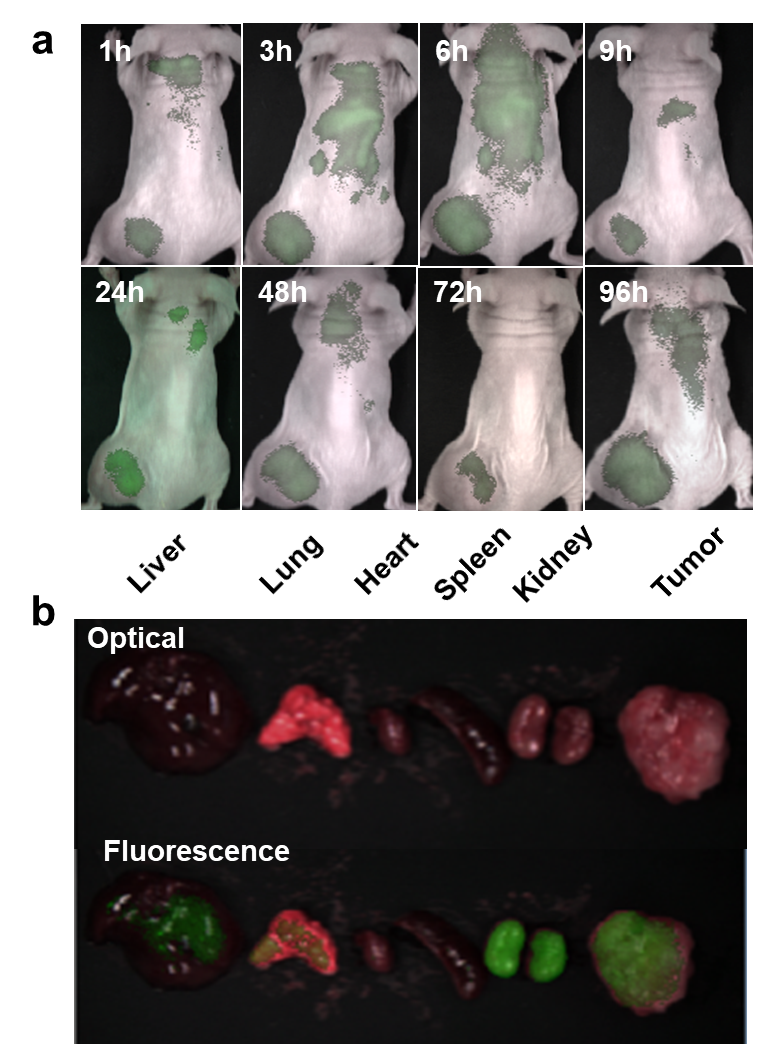


**Figure S2.** Biodistribution of ICG-CNPs in VX2 tumor-bearing mouse. **a**) Non-invasive in vivo NIRF images of the whole body over time. **b**) Ex vivo NIRF image of the main organs (liver, spleen, lungs, kidneys, and heart) and tumor at 96 h post-injection.


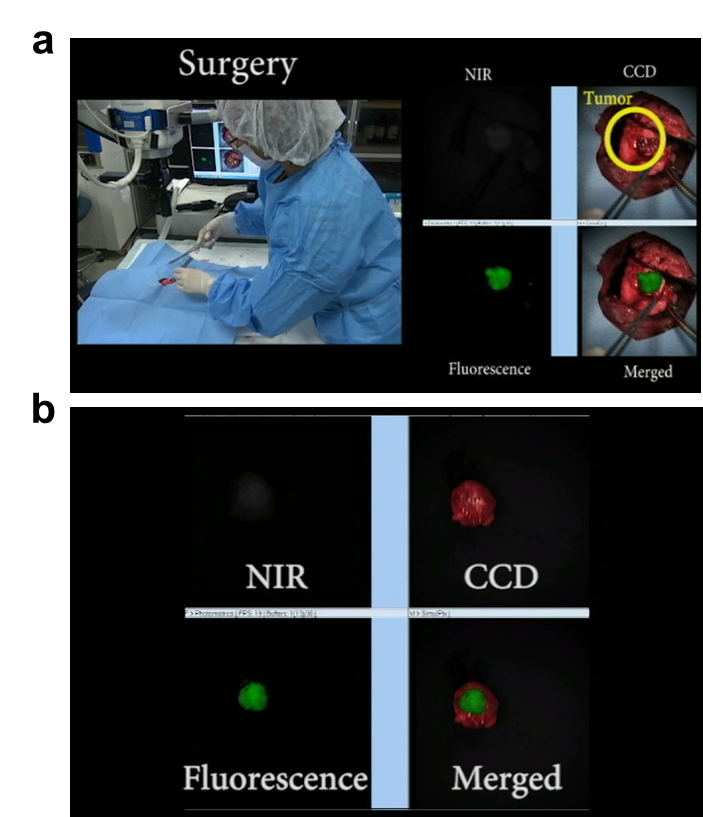


**Figure S3.** Image-guided surgical removal of orthotopic VX2 lung tumor in rabbit models. **a**) Real-time NIRF image guided surgical removal of tumor tissue. **b**) Excised tumor tissue from lung tissue.
